# Using social media in health literacy research: A promising example involving Facebook with young Aboriginal and Torres Strait Islander males from the Top End of the Northern Territory

**DOI:** 10.1002/hpja.421

**Published:** 2020-10-19

**Authors:** James A. Smith, Anthony Merlino, Ben Christie, Mick Adams, Jason Bonson, Richard H. Osborne, Murray Drummond, Barry Judd, David Aanundsen, Jesse Fleay, Himanshu Gupta

**Affiliations:** ^1^ Freemasons Centre for Male Health & Wellbeing ‐ Northern Territory Menzies School of Health Research Casuarina NT Australia; ^2^ Charles Darwin University Darwin NT Australia; ^3^ Edith Cowan University Joondalup WA Australia; ^4^ Northern Territory Department of Health Darwin NT Australia; ^5^ Swinburne University of Technology Hawthorn VIC Australia; ^6^ Flinders University Adelaide SA Australia; ^7^ University of Melbourne Melbourne VIC Australia; ^8^ Fred Hollows Foundation Casuarina NT Australia

## 1 INTRODUCTION

This brief report describes three key lessons learned during a health literacy research project with young Aboriginal and Torres Strait Islander males from the Top End of the Northern Territory (NT), Australia. More specifically, it is a methodologically focused paper that discusses processes associated with using a combination of yarning sessions and social media content as tools to unpack conceptualisations of health and well‐being among this marginalised population. The lessons discussed include (a) the utility of using social media in providing an authentic window into the lives of a hard‐to‐reach populations; (b) the need to carefully consider ethical implications; and (c) the benefits of using social media content to triangulate data and enhance methodological rigour. To understand the methodological contribution social media can make to equity‐focused health literacy research, it is first useful to understand what is meant by health literacy.

Globally, the term ‘health literacy’ has been adopted widely, and defined broadly, in a range of health promotion policy, practice and research contexts.^1^, ^2^, ^3^, ^4^, ^5^, ^6^, ^7^ This has extended to concepts such as health literacy responsiveness and distributed health literacy.^6^, ^7^ A focus on health literacy measurement has been a significant part of the emerging health literacy discourse.^8^, ^9^, ^10^, ^11^ This has focused on ways to measure health literacy at both individual and population level.^5^ For example, popular and well tested tools developed by Australian researchers have included the Health Literacy Questionnaire (HLQ), Information and Support for Health Action – Questionnaire (ISHA‐Q) and the Conversational Health Literacy Assessment Tool (CHAT).^12^, ^13^, ^14^ These tools, and others, have increasingly been used in contexts with vulnerable populations where health inequities are well documented,^13^, ^15^, ^16^, ^17^ including Aboriginal and Torres Strait Islander people.^17^, ^18^ However, multiple scholars have also pointed toward the importance of adopting qualitative approaches to better understand the health literacy needs of vulnerable and marginalised populations, including Aboriginal and Torres Strait Islander populations.^18^, ^19^

Qualitative research approaches, when applied to health literacy research, can assist the health promotion community to better tailor programs and policy responses that aim to reduce health inequities among marginalised populations.^5^, ^19^, ^20^ For example, yarning has been increasingly used to understand the health and well‐being needs of Aboriginal and Torres Strait Islander people at individual, family and community levels.^18^, ^20^, ^21^ Yarning typically involves a fluid and interactive discussion with participants in a culturally safe environment. Interviews and focus groups have also been used repeatedly in health literacy research with youth, migrant, and low socioeconomic status populations.^22^, ^23^, ^24^ Visual methods, such as Photovoice, have also been increasingly used in health literacy research – primarily with youth, men and people with mental health conditions.^25^, ^26^, ^27^ Photovoice is a community‐based research method that involves capturing a participants’ ideas and reality on a given topic through photographs, and then discussing these through group interviews or individual interviews.^28^, ^29^ These types of visual methods are perceived to be a powerful means for minority populations to express their understandings of health and well‐being.^29^ It can support critical thinking, self‐reflection, discovering strengths and social support.^28^ However, there are also important ethical implications that need careful consideration when using visual research methods in health promotion practice and research contexts. These relate to a broad range of recruitment, consent, engagement, use and research translation issues.^30^, ^31^, ^32^

### 1.1 Approach

In this brief report, we offer new methodological and ethical insights about the way popular and emerging social media platforms can be used in equity‐focused health literacy research. More specifically, we reflect on our experiences of using Facebook to gain a deeper understanding of the health literacy needs of young Aboriginal and Torres Strait Islander males in the Top End of the Northern Territory, Australia.^18^ This project received ethics approval from the Charles Darwin University Human Research Ethics Committee (H18043). The intent of this paper is not to present empirical findings. Rather, we aim to discuss the methodological and ethical benefits of using social media platforms – such as Facebook, Snapchat, Instagram and Tik Tok – to undertake content analyses in health literacy research. We present three lessons learned when using Facebook in this way, but consider the concepts could be extended to other social media platforms, particularly those use by marginalised or vulnerable populations. We envisage these insights will make a valuable contribution to professional dialogue and debate on this topic.

### 1.2 Lessons learned from health literacy research involving social media

#### 1.2.1 Social media can provide an authentic window into the lives of ‘hard‐to‐reach’ populations

A significant research base suggests that vulnerable populations experiencing health inequities can be difficult to engage through health promotion research and practice.^33^, ^34^, ^35^ Evidence points toward strategies that involve meeting these ‘hard‐to‐reach’ populations on their terms in settings and environments of relevance to them.^33^, ^34^, ^35^ In our case, we were interested in the lived‐experiences of young Aboriginal and Torres Strait Islander males. Current scholarship suggests that both youth and men are considered to be hard‐to‐reach populations in health research contexts.^36^, ^37^ Emerging evidence also indicates that youth are actively using social media to communicate with their social networks, with some scholars arguing that social media has become their virtual world.^38^

During yarning sessions with young males, we observed that participants were readily accessing and using a range of social media platforms, including Facebook. After discussing this with the Chair of our Human Research Ethics Committee, we successfully sought an ethics amendment to approach the yarning session participants to seek their informed consent to access their Facebook posts and related conversation threads. We limited this to retrospective posts and threads from the last two years – that is, information that had been posted prior to the date of consent. Our original intent was to gauge the extent to which participants discussed health and well‐being issues through social media. Our early analysis revealed there was significant content being posted that related to their health and well‐being, and that of their friends and family. Importantly, these posts reflected a pre‐existing and authentic expression of their day‐to‐day lives. This contrasts many other qualitative research approaches where information is sought, and thus generated, for the purpose of further analysis.

**FIGURE 1:**
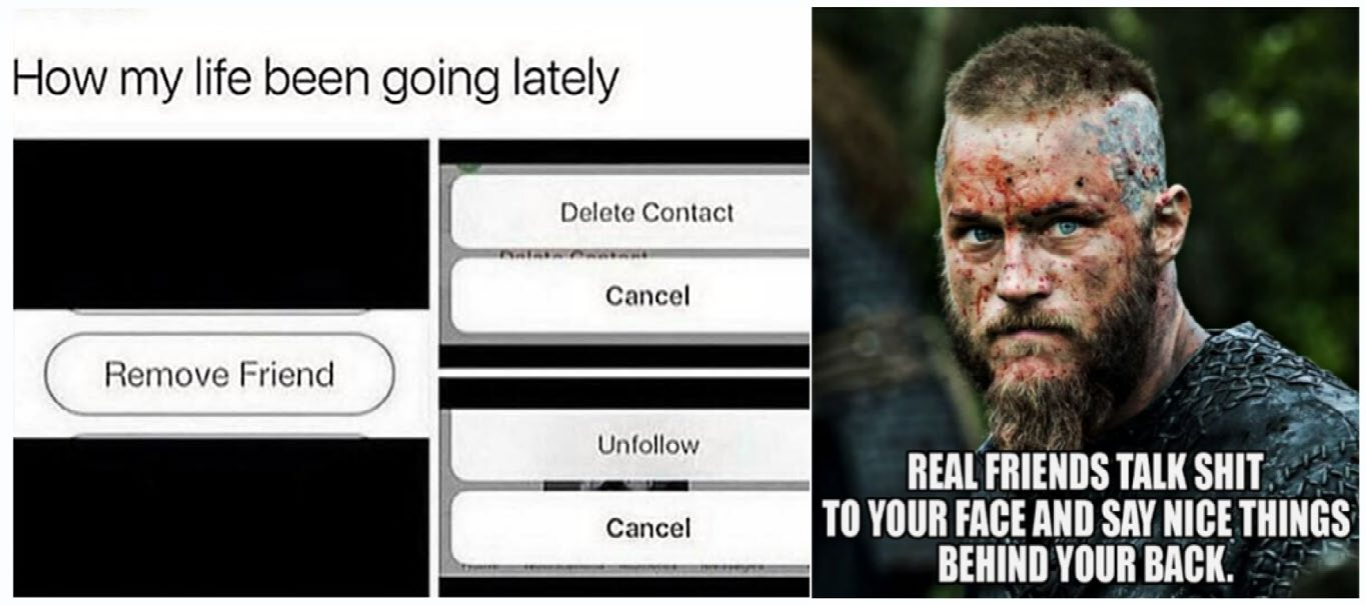
Example meme about friendship [Colour figure can be viewed at wileyonlinelibrary.com]

**FIGURE 2:**
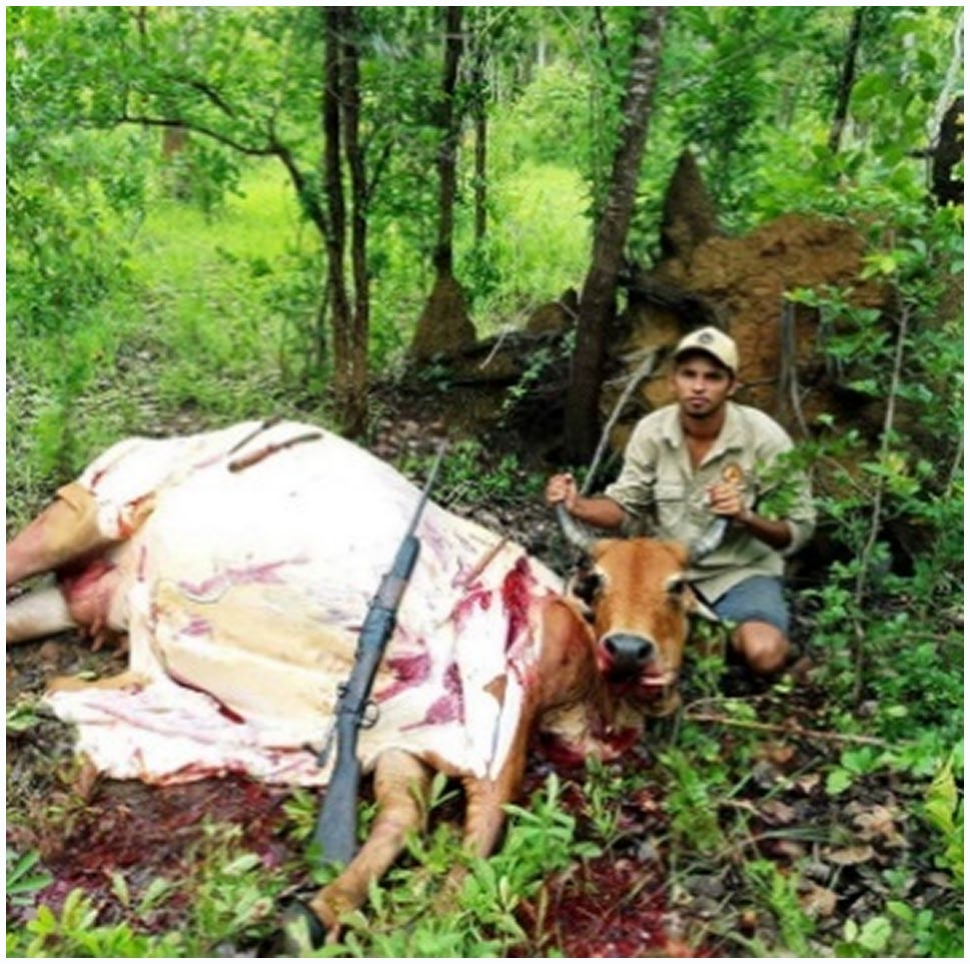
Example image of engagement of on‐country activities (eg hunting) [Colour figure can be viewed at wileyonlinelibrary.com]

**FIGURE 3:**
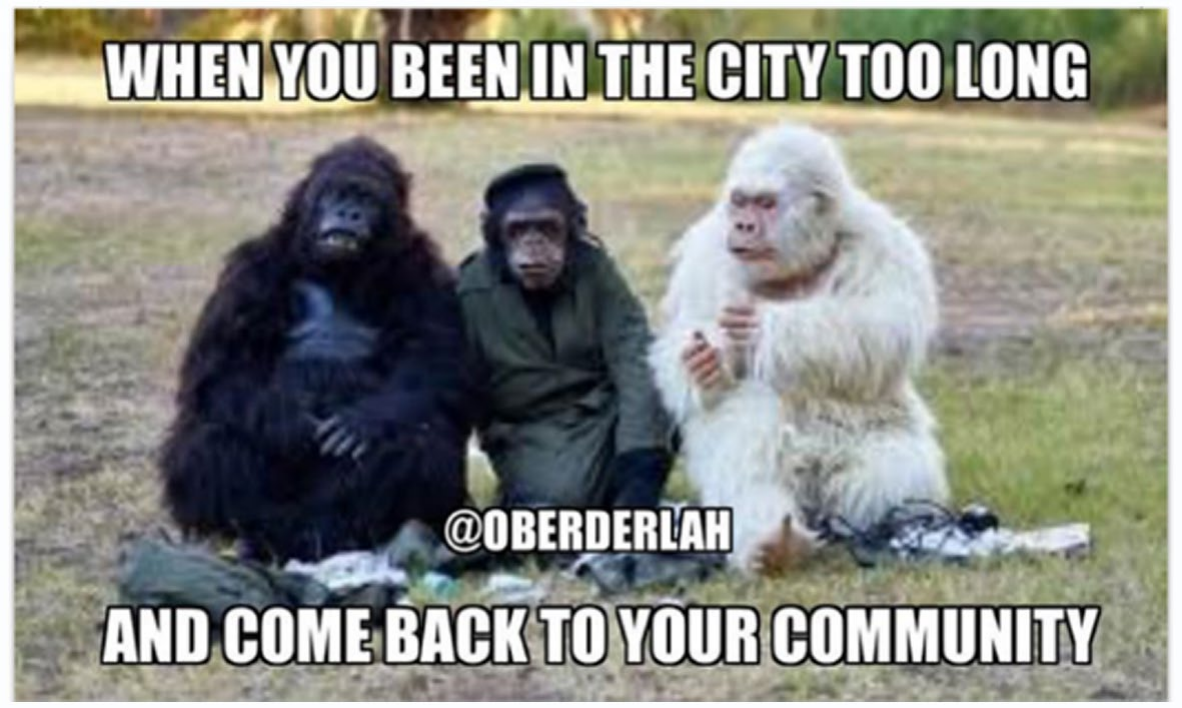
Example meme of identify formation [Colour figure can be viewed at wileyonlinelibrary.com]

**FIGURE 4:**
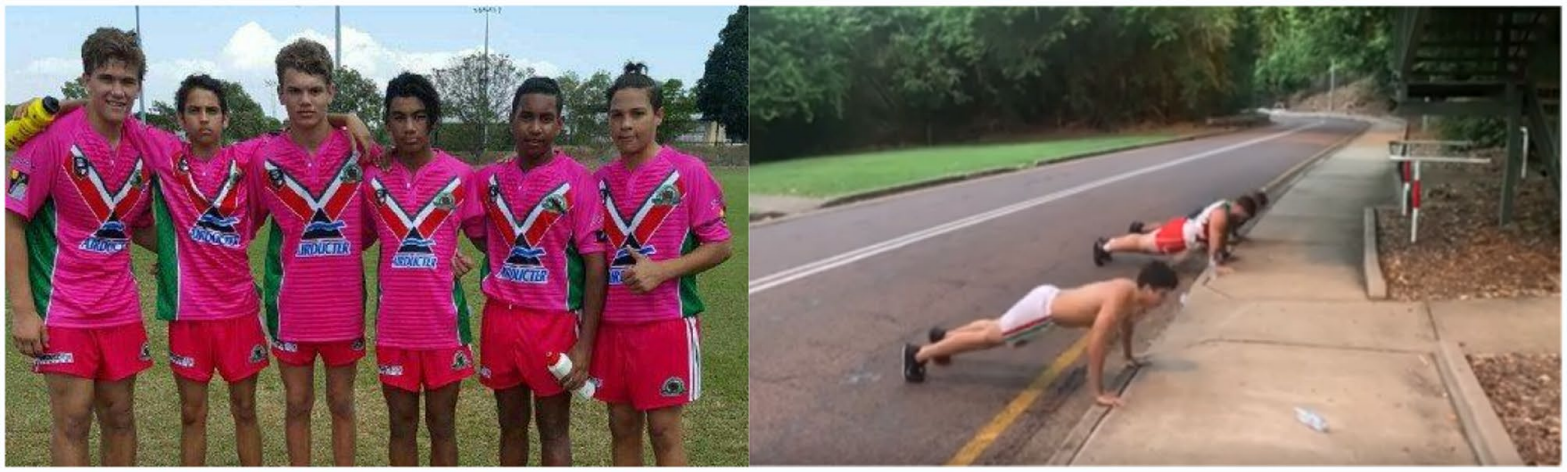
Example images of participation in team sports and physical activity [Colour figure can be viewed at wileyonlinelibrary.com]

**FIGURE 5:**
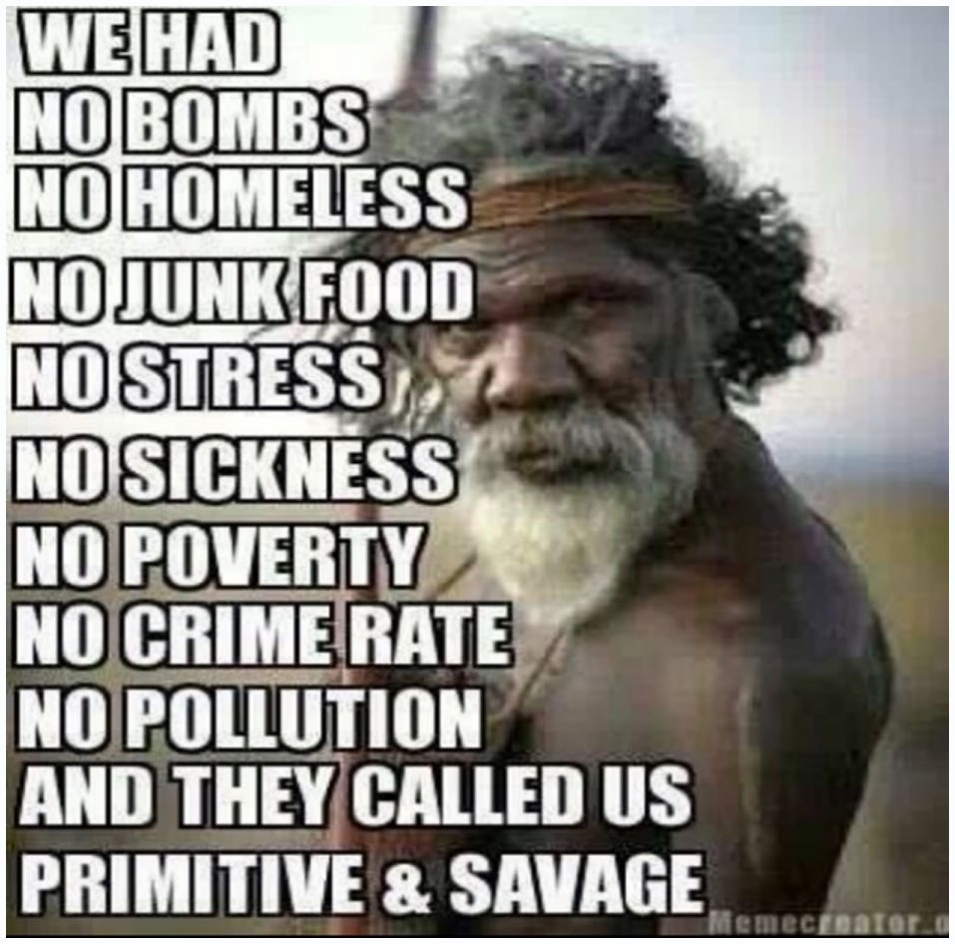
Example meme about reflections on the social determinants of health [Colour figure can be viewed at wileyonlinelibrary.com]

We recognise that Facebook posts may not be a true and accurate reflection of an individual's health behaviours – that is, there may be a gap between expressed health perceptions and attitudes, and subsequent health behaviours. Indeed, some researchers have suggested that social media evokes unique styles of public performance that can differ markedly from non‐virtual behaviours.^39^ This is particularly relevant in the context of scholarship indicating a rapid breakdown of public‐private spheres of life. Nevertheless, we argue that Facebook provides authentic and non‐invasive insights into the way youth are communicating to their peers about health and well‐being issues. Within the context of our research this included images and memes about friendship (Figure 1); engagement in on‐country activities such as hunting (Figure 2); identity formation (Figure 3); participation in team sports and physical activity (Figure 4); and reflections about the social determinants of health (Figure 5). The Facebook content analysis also enabled us to learn about other determinants of health literacy, such as the impacts of gender, racism, employment, education, housing and incarceration. A more detailed empirical analysis of this research, including an analysis of yarning session data and Facebook content, has been published elsewhere.^40^

These qualitative perspectives are unique, and paying attention to these Facebook discussions can offer the health promotion community important insights about the health literacy of hard‐to‐reach populations, with potential to inform needs assessment, planning and evaluation practices that are concordant with core health promotion competencies.

#### 1.2.2 Careful consideration of ethical implications is important

Two critical ethical implications that we were required to navigate in our study related to that of informed consent; and researcher burden.

In our research with young Aboriginal and Torres Strait Islander males, the staging of different research phases meant that seeking informed consent was a relatively easy process. That is, when participants were involved in a Yarning Session, we sought either written or oral informed consent at that point in time to enable access to their personal Facebook page, and to make use of the retrospective content they had shared. This included the ability to access and use their commentary, photos and memes. We explicitly explained that this was for research analysis and research translation purposes to improve our understanding of health literacy that could be used to inform future health policy and practice endeavours, and thus have a benefit to other young Aboriginal and Torres Strait Islander males. This was generally met with a high level of enthusiasm. While this process was relatively straightforward, many of the Facebook posts included images and commentary of friends and family. This was inherently important to the research topic, as it indicated the importance of support structures – such as friends and family – in the way health and well‐being was negotiated among this demographic, and was indicative of the perceived influence of those people providing health advice. However, in this instance, we had not received a form of secondary consent from people whose images had been uploaded by the participant, nor had we sought the consent from people who had made a contribution to the Facebook feeds of our participants. This created an ethical conundrum. At this juncture, it is important to mention it was not a requirement of our ethics approval to seek secondary consent. Yet, had we been using traditional photovoice methods, this would have been an expectation.

We argue that secondary consent – in an era of endless ‘selfies’ (where people take photos of themselves – often with friends and family), and with an increasing uptake of multiple social media platforms that are based on sharing visual images, particularly among youth – is a complex issue within the realm of research utilising social media. We have entered a terrain where ethical expectations (particularly those which existed prior to the rapid global advancement of social media) are no longer concordant with public expectations. That is, expectations about the ways in which the risks and benefits are assessed in health promotion research involving social media – including seeking secondary consent – need to be constantly reviewed in line with the social and cultural values of the population under investigation. In our case it was evident that young Aboriginal and Torres Strait Islander males were readily using Facebook and other social media platforms, and seldom sought secondary consent from their friends when posting their images on Facebook in their everyday lives. More often than not these were public posts – effectively meaning these are considered publicly accessible documents. Therefore, exemptions for secondary consent may be plausible for research that is interested in the everyday lives of vulnerable populations, such as Aboriginal and Torres Strait Islander males. However, this is a contentious issue, whereby ethical protocols and guidelines used by Human Research Ethics Committees, alongside principles for guiding Indigenous research, sometimes lack clarity about these blurred‐lines. This is particularly problematic where accepted social media practices and emerging social media platforms are evolving at a pace much faster than that of revision processes associated with research ethics guidelines and principles. When adopting content analysis through social media platforms, significant effort must, therefore, be invested in understanding and appreciating the cultural and social context of the population under consideration, and how these contextual factors generate unique social media practices. These ethical issues may become increasingly complicated in situations where voluntary informed consent is sought virtually, rather than in person. This observation also has significant implications for publishing research findings that have used social media platforms to inform data analysis, as some publishers are now also requiring evidence of consent procedures. In our experience, publisher expectations associated with informed consent may well differ to those approved by certified human research ethics committees, which can create additional ethical dilemmas.

We recognise there is a burgeoning body of evidence discussing the ethical implications of using social media, including Facebook, in public health research contexts.^36^, ^41^ This extends to strategies associated with recruitment,^36^, ^37^, ^41^ health education,^42^, ^43^ information sharing,^42^, ^44^ social marketing^43^ and content analysis.^37^, ^44^ Necessarily, this involves careful navigation of ethical considerations, such as negotiating informed consent. However, it also involves consideration of the imposed research burden. Indeed, the research burden placed upon study participants is an important consideration in any health promotion research. This is particularly relevant for Aboriginal and Torres Strait Islander research, where research participation burden has been particularly problematic in the past.^45^, ^46^ We argue that the retrospective use and analysis of Facebook content significantly reduces the participant research burden, when compared to other qualitative data collection methods such as interviews, focus groups and traditional forms of photovoice. Indeed, in our research there was no additional research burden placed upon participants, other than seeking informed consent during yarning sessions. This makes Facebook analyses an attractive, time‐efficient and cost‐effective health promotion research tool.

#### 1.2.3 Analysing social media content can be a useful way to triangulate data

Data or methodological triangulation is an important approach used in qualitative health research internationally.^47^ This involves comparing and contrasting one source of data to another to increase the validity of assumptions made.^47^, ^48^ In this sense, data triangulation is a way to strengthen the legitimacy and rigour of research findings. In our research, we used Facebook data to test the validity and generalisability of findings from the thematic analysis of nine yarning sessions with 37 young Aboriginal and Torres Strait Islander males. The yarning sessions aimed to examine participant understandings about health literacy, with subsequent discussion about personal and professional relationships; health information access and comprehension; health attitudes and behaviours; life aspirations; and health program and service environments.^40^ Similar concepts were used as a lens to analyse the content of Facebook posts. While the research team did not ask participants about their Facebook posts – primarily due to time constraints – we still consider this methodological triangulation to be beneficial. By analysing Facebook content, and comparing this with yarning session data, we were more confident in the themes we had identified, in addition to identifying different themes and sub‐themes that did not emerge during yarning sessions. For example, discussion and images reflecting sovereignty were evident in Facebook posts, which did not surface in the yarning sessions. In summary, Facebook posts are a readily available source of data that can benefit research with vulnerable populations.

## 2 CONCLUSION

In this brief report we have discussed how one social media platform, Facebook, can be a useful source of information – particularly when used in conjunction with other methods – to ascertain broader understandings of health literacy among a marginalised population in Australia. We have described how Facebook provides an authentic perspective into the lives of hard‐to‐reach populations; requires considered and pragmatic thought about ethical considerations such a secondary consent and participant research burden; and can be used strategically as a data triangulation tool in qualitative health research. We recognise this paper only provides a snapshot into the utility of Facebook in health literacy research with vulnerable populations. It is important to reiterate that while we observed that participants were readily accessing and using a range of social media platforms, the subsequent interaction has provided a valuable contribution toward ongoing professional dialogue associated with engaging young Aboriginal and Torres Strait Islander males in health promotion research. Retrospectively, this professional dialogue has guided further thinking about constructive and positive ways to engage young Aboriginal and Torres Strait Islander males in discussion about their health and well‐being through social media, including those involving peers, family and community. We would encourage health promotion researchers, policymakers and practitioners to engage in further debate about the ongoing challenges and opportunities of such approaches. We anticipate the dynamics shaping other social media platforms may differ to those we have encountered with Facebook. As such, we encourage researchers with an interest in equity and health literacy research to explore how other social media platforms can also be used innovatively to understand and respond to the unique health and social needs of maginalised populations.

## References

[1] Nutbeam D . The evolving concept of health literacy. Soc Sci Med. 2008;67(12):2072–8. 18952344 10.1016/j.socscimed.2008.09.050

[2] Peerson A , Saunders M . Health literacy revisited: What do we mean and why does it matter? Health Promot Int. 2009;24(3):285–96. 19372101 10.1093/heapro/dap014

[3] Sørensen K , Van den Broucke S , Fullam J , Doyle G , Pelikan J , Slonska Z , et al. Health literacy and public health: A systematic review and integration of definitions and models. BMC Public Health. 2012;12(80):80–93. 22276600 10.1186/1471-2458-12-80 PMC3292515

[4] Bröder J , Okan O , Bauer U , Bruland D , Schlupp S , Bollweg TM , et al. Health literacy in childhood and youth: a systematic review of definitions and models. BMC Public Health. 2017;17(1):361–86. 28441934 10.1186/s12889-017-4267-y PMC5405535

[5] Smith J , Ireland S . Towards equity and health literacy. Health Promot J Austr. 2020;31(1):3–4. 31903649 10.1002/hpja.317

[6] Trezona A , Dodson S , Osborne R . Development of the organisational Health Literacy Responsiveness (Org‐HLR) self‐assessment tool and process. BMC Health Serv Res. 2018;18:694. 30189874 10.1186/s12913-018-3499-6 PMC6128002

[7] Ewards M , Wood F , Davies M , Edwards A . ‘Distributed health literacy’: longitudinal qualitiative analysis of the roles of health loiteracy mediators and social networks of people with living long‐term health conditions. Health Exp. 2015;18(5):1180–93. 10.1111/hex.12093 PMC5060848 23773311

[8] Jordan J , Buchbinder R , Osborne R . Conceptualising health literacy from the patient perspective. Patient Educ Couns. 2010;70(1):36–42. 10.1016/j.pec.2009.10.001 19896320

[9] Pleasant A , McKinney J , Rikard R . Health literacy measurement: a proposed research agenda. J Health Commun. 2011;16(S3):11–21. 21951240 10.1080/10810730.2011.604392

[10] Bann C , McCormack L , Berkman N , Squiers L . The health literacy skills instrument: a 10‐item short form. J Health Commun. 2012;17(S3):191–202. 23030570 10.1080/10810730.2012.718042 PMC12051260

[11] Guzys D , Kenny A , Dickson‐Swift V , Threlkeld G . A critical review of population health literacy assessment. BMC Public Health. 2015;15:215–22. 25885742 10.1186/s12889-015-1551-6 PMC4351936

[12] Osborne R , Batterham R , Elsworth G , Hawkins M , Buchbinder R . The grounded psychometric development and initial validation of the Health Literacy questionnaire (HLQ). BMC Public Health. 2013;13(658):1–17. 23855504 10.1186/1471-2458-13-658 PMC3718659

[13] Batterham R , Hawkins M , Collins P , Buchbinder R , Osborne R . Health literacy: applying current concepts to improve health services and reduce health inequalities. Public Health. 2016;132:3–12. 10.1016/j.puhe.2016.01.001 26872738

[14] O’Hara J , Hawkins M , Batterham R , Dodson S , Osbrone R , Beauchamp A . Conceptualisation and development of the Conversational Health Literacy Assessment Tool (CHAT). BMC Health Serv Res. 2018;18(199):1–8. 29566755 10.1186/s12913-018-3037-6 PMC5863801

[15] Logan RA , Wong WF , Villaire M , Daus G , Parnell TA , Willis E , et al. Health literacy: a necessary element for achieving health equity. NAM Perspectives. Discussion Paper. Washington, DC: National Academy of Medicine; 2015.

[16] Muscat D , Smith S , Dhillon H , Morony S , Esther D , Shepherd H , et al. Incorporating health literacy in education for socially disadvantaged adults: an Australian feasibility study. Int J Equity Health. 2016;15:1–10. 27259476 10.1186/s12939-016-0373-1 PMC4893249

[17] Rheault H , Coyer F , Jones L , Bonner A . Health literacy in Indigenous people with chronic disease living in remote Australia. BMC Health Serv Res. 2019;19:523–33. 31349842 10.1186/s12913-019-4335-3 PMC6659262

[18] Smith J , Christie B , Bonson J , Adams M , Osbrone R , Judd B , Drummond M , Aanundsen D , Fleay J . Health Literacy Among Young Aboriginal and Torres Strait Islander Males in The Northern Territory. Darwin, Australia: Menzies School of Health Research; 2019. 10.1002/hpja.421 PMC7984039 32946620

[19] Boot G , Lowell A . Acknowledging and promoting Indigenous knowledges, paradigms and practices within health literacy‐related policy and practice documents across Australia, Canada and New Zealand. Int Indig Policy J. 2019;10(3):1–28.

[20] Bessarab D , Ng’Andu, B . Yarning about yarning as a legitimate method in Indigenous research. Int J Crit Indig Stud. 2010;3(1):37–50.

[21] Vujcich D , Lyford M , Bellottie C , Bessarab D , Thompson S . Yarning quiet ways: Aboriginal carers’ views on talking to youth about sexuality and relationships. Health Promot J Austr. 2017;29(1):39–45. 29700932 10.1002/hpja.14

[22] Okan O , Bollweg T , Broder J , Pinheiro P , Bauer U . Qualitative methods in health literacy research in young children. Eur J Pub Health. 2017;27(S3):54.

[23] Santos M , Gorukanti A , Jurkunas L , Handley M . The health literacy of U.S. immigrant adolescents: a neglected research priority in a changing world. Int J Environ Res Public Health. 2018;15(10):1–18. 10.3390/ijerph15102108 PMC6209953 30257475

[24] Aldin A , Chakraverty D , Baumeister A , Monsef I , Noyes J , Jakob T , Seven ÜS , Anapa G , Woopen C , Kalbe E , Skoetz N . Gender differences in health literacy of migrants: a synthesis of qualitative evidence. Cochrane Database Syst Rev. 2019;2019(4):CD013302. 10.1002/14651858.CD013302.pub2 PMC11635922 39665382

[25] Oliffe J , Bottorff J . Further than the eye can see? Photo elicitation and research with men. Qual Health Res. 2007;17(6):850–8. 17582026 10.1177/1049732306298756

[26] Han C , Oliffe J . Photovoice in mental illness research: A review and recommendations. Health. 2016;20(2):110–26. 25673051 10.1177/1363459314567790 PMC4768711

[27] Creighton G , Brussoni J , Oliffe J , Han C . Picturing masculinities: Using photo‐elicitation in men’s health research. Am J Mens Health. 2017;11(5):1472–85. 26483294 10.1177/1557988315611217 PMC5675207

[28] Velez‐Grau C . Using Photovoice to examine adolescents’ experiences receiving mental health services in the United States. Health Promot Int. 2018;35(5):912–20. 10.1093/heapro/day043 29986026

[29] Halvorsrud K , Rhodes J , Webster G , Francis J , Haarmans M , Dawkins N , et al. & Mental Health Organisations. Photovoice as a promising public engagement approach: capturing and communicating ethnic minority people’s lived experienced of severe mental illness and its treatment. BMJ Open Qual. 2009;8(4):1–5. 10.1136/bmjoq-2019-000665 PMC6863667 31798067

[30] Wang C , Redwood‐Jones Y . Photovoice ethics: perspectives from Flint Photovoice. Health Educ Behav. 2011;28(5):560–72. 10.1177/109019810102800504 11575686

[31] Hannes K , Parylo O . Let’s play it safe: ethical considerations from participants in Photovoice research project. Int J Qual Methods. 2014;13:255–74.

[32] Creighton G , Oliffe J , Olivier F , Bottorff J , Broome A , Jenkins E . Photovoice ethics: reflections from men’s mental health research. Qual Health Res. 2017;28(3):446–55. 28962540 10.1177/1049732317729137 PMC5764141

[33] Shaghaghi A , Bhopal R , Sheikh A . Approaches to recruiting ‘hard‐to‐reach’ populations into research: a review of the literature. Health Promot Perspect. 2011;1(2):86–94. 24688904 10.5681/hpp.2011.009 PMC3963617

[34] Bonevski B , Randell M , Paul C , Chapman K , Twyman L , Bryant J , et al. Reaching the hard‐to‐reach: a systematic review of strategies for improving health and medical research with socially disadvantaged groups. BMC Med Res Methodol. 2014;14:42. 24669751 10.1186/1471-2288-14-42 PMC3974746

[35] Ellard‐Gray A , Jeffrey N , Choubak M , Crann S . Finding the hidden participant: solutions for recruiting hidden, hard‐to‐reach, and vulnerable populations. Int J Qual Methods. 2015;14:1–10.

[36] Amon K , Campbell A , Hawke C , Steinbeck K . Facebook as a recruitment tool for adolescent health research: a systematic review. Acad Pediatr. 2014;14(5):439–47. 25169155 10.1016/j.acap.2014.05.049

[37] Sendall M , McCokser L , Crane P , Rowland B , Fleming M , Briggs H . Using Facebook for health promotion in “hard‐to‐reach” truck drivers: qualitative analysis. J Med Internet Res. 2018;20(11):e286. 30389653 10.2196/jmir.9689 PMC6238102

[38] Sclater M , Lally V . The realities of researching alongside virtual youth in late modernity creative practices and activity theory. J Youth Studies. 2014;17(1):1–25.

[39] Neiger B , Thackeray R , Van Wagenen S . Use of social media in health promotion: purposes, key performance indicators, and evaluation metrics. Health Prom Pract. 2012;13(2):159–64. 10.1177/1524839911433467 22382491

[40] Authors withheld for blind review . “Dudes are meant to be tough as nails”: The nexus between masculinities, culture, and health literacy from the perspective of young Aboriginal and Torres Strait Islander males – Implications for policy and practice. Am J Mens Health. 2020;14(3):1557988320936121. 32583723 10.1177/1557988320936121 PMC7318825

[41] Whitaker C , Stevelink S , Fear N . The use of Facebook in recruiting participants for health research purposes. A systematic review. J Med Internet Res. 2017;19(8):e290. 28851679 10.2196/jmir.7071 PMC5594255

[42] Syred J , Naidoo C , Woodhall S , Baraitser P . Would you tell everyone this? Facebook conversations as health promotion interventions. J Med Internet Res. 2014;16(4):e108. 24727742 10.2196/jmir.3231 PMC4042608

[43] Kite J , Foley B , Grunseit A , Freeman B . Please like me: Facebook and public health communication. PLoS One. 2016;11(9):e0162765. 27632172 10.1371/journal.pone.0162765 PMC5025158

[44] Sharma M , Yadav K , Yadav N , Ferdinand K . Zika virus pandemic: analysis of Facebook as a social media health information platform. Am J Infect Control. 2017;45(3):301–2. 27776823 10.1016/j.ajic.2016.08.022

[45] Dudgeon P , Kelly K , Walker R . Closing the gaps in and through Indigenous health research: guidelines, processes and practices. Aust Aborig Stud. 2010;2:81–91.

[46] Thomas D , Bainbridge R , Tsey K . Changing discourses in Aboriginal and Torres Strait Islander health research, 1914–2014. Med J Aust. 2014;201(1):S1–4. 10.5694/mja14.00114 25047769

[47] Bekhet A , Zauszniewski J . Methodological triangulation: an approach to understanding data. Nurse Res. 2012;20(2):40–3. 23316537 10.7748/nr2012.11.20.2.40.c9442

[48] Carter N , Bryant‐Lukosius D , DiCenso A , Blythe J , Neville A . The use of triangulation in qualitative research. Oncol Nurs Forum. 2014;41(5):545–7. 25158659 10.1188/14.ONF.545-547

